# Long non-coding SBF2-AS1 acting as a competing endogenous RNA to sponge microRNA-142-3p to participate in gemcitabine resistance in pancreatic cancer via upregulating TWF1

**DOI:** 10.18632/aging.102307

**Published:** 2019-10-31

**Authors:** Yong-Qiang Hua, Yao-Dong Zhu, Guo-Qun Xie, Ke Zhang, Jie Sheng, Zhen-Feng Zhu, Zhou-Yu Ning, Hao Chen, Zhen Chen, Zhi-Qiang Meng, Lu-Ming Liu

**Affiliations:** 1 Minimally Invasive Treatment Center, Fudan University Shanghai Cancer Center, Shanghai 200032, PR China; 2 Department of Oncology, Shanghai Medical College, Fudan University, Shanghai 200032, PR China; 3 Chinese Integrative Medicine Oncology Department, First Affiliated Hospital of Medical University of Anhui, Hefei 230000, Anhui Province, PR China; 4 Oncology Department, Yueyang Hospital of Integrative Chinese and Western Medicine Affiliated to Shanghai University of Traditional Chinese Medicine, Shanghai 200437, PR China

**Keywords:** long non-coding SBF2-AS1, competing endogenous RNA, microRNA-142-3p, gemcitabine resistance, pancreatic cancer

## Abstract

Objective: This study is implemented to probe into the function of lncRNA SBF2-AS1 as a competing endogenous RNA (ceRNA) to sponge microRNA-142-3p (miR-142-3p) in modulating TWF1 expression in the gemcitabine resistance of pancreatic cancer.

Results: LncRNA SBF2-AS1 was highly expressed in pancreatic cancer tissues and cells. SBF2-AS1 was found to be associated with gemcitabine resistance in pancreatic cancer. Knock-down of SBF2-AS1 inhibited proliferation, epithelial-mesenchymal transition, while promoting apoptosis of gemcitabine resistant pancreatic cancer cells. SBF2-AS1 inhibited the expression of TWF1 by competitively binding with miR-142-3p in pancreatic cancer.

Conclusion: Our study demonstrates that knock-down of SBF2-AS1 inhibits the expression of TWF1 by competitively binding with miR-142-3p to induce gemcitabine resistance in pancreatic cancer.

Methods: Expression of SBF2-AS1 was tested in pancreatic cancer tissues and cells. Construction of AsPC-1/GEM and PANC-1/GEM cells with low expression of SBF2-AS1 was performed to determine the biological behaviors of drug-resistant cells. AsPC-1 and PANC-1 cells expressing SBF2-AS1 and/or miR-142-3p were constructed and treated with different concentrations of gemcitabine to detect the sensitivity of the cells to gemcitabine. The binding relationship between SBF2-AS1 and miR-142-3p and between miR-142-3p and TWF1 were determined.

## INTRODUCTION

Pancreatic cancer is a main health problem in industrialized countries, which represents the 4^th^ reason of cancer-related death worldwide [1, 2]. It is estimated that approximately 90% of patients suffering from pancreatic cancer are caused by environmental risk factors, and approximately 50% of them may be attributed to diet [3]. The treatment of pancreatic cancer is based upon a multidisciplinary approach which contains surgery, radiotherapy and chemotherapy, even if the effect of therapy is merely palliative [4]. Since 1997, gemcitabine therapy has been considered as the first-line treatment for those patients who suffered from unresectable locally metastatic or advanced pancreatic cancer [5]. For the patients with metastatic pancreatic cancer, the five-year survival rate is only 2%, and the one-year survival rates are 17 to 23% with the treatment of gemcitabine [6]. Based on this, developing new and effective therapeutic regimens is important for alleviating the prognosis of pancreatic cancer patients.

Long non-coding RNAs (lncRNAs) are a class of measurably conserved and polyadenylated ncRNAs, and they have vital roles in tumorigenesis [7]. Additionally, lncRNAs are suggested to be implicated in pancreatic cancer, which could be a useful biomarker in the prognostic prediction and treatment of pancreatic cancer [8]. Among which, lncRNA SBF2-AS1 has been revealed to act a regulator in tumor progression. For instance, a prior study found out that SBF2-AS1 is highly expressed in non-small cell lung cancer (NSCLC), which contributes to the promotion of proliferation of NSCLC [9, 10]. Zhang et al. have stated that SBF2-AS1 promoted metastasis of hepatocellular carcinoma via controlling epithelial-mesenchymal transition (EMT) and forecasted unfavorable prognosis [11]. Chen et al. have stated that high expression of SBF2-AS1 was found in esophageal squamous cell carcinoma (ESCC), and silenced SBF2-AS1 repressed proliferative and invasive ability of ESCC cells [12]. It has also been elucidated that SBF2-AS1 suppression restricted cervical cancer cells proliferation [13]. Nevertheless, the underlying mechanism of SBF2-AS1 remains unclear in pancreatic cancer. Recently, lncRNAs are demonstrated to be implicated in tumor progression via functioning as competing endogenous RNAs (ceRNAs) for miRNAs [14]. Some studies with the application of microarrays have demonstrated that abnormal expressed microRNAs (miRNAs) in pancreatic ductal adenocarcinoma (PDAC) have a great influence on coding-gene expression [15–18]. Recently, Tang et al. have supported that miR-142-3p expression was decreased relative to the normal ones, suggesting a regulatory role of miR-142-3p in cervical cancer [19]. miR-142-3p has been also mirrored to attenuate stem cell characteristics of breast cancer and reduces radioresistance *in vitro* [20]. Meanwhile, some recent studies also suggested that miR-142-3p is capable of restricting cell proliferation and chemoresistance in ovarian cancer, human osteosarcoma, and PDAC via targeting different target genes [21–24]. The cytoskeleton genes twinfilin 1 (TWF1), also called PTK9, was elucidated to modulate drug sensitivity along with cancer progression [25]. Besides, TWF1 has been shown to exclusively function as an actin-monomerse-questering protein [26]. Kaishang et al. have found that [27] robustness and poor prognosis in Lung adenocarcinoma (LUAD) associated with TWF1 levels thus making it a appropriate therapeutic biomarker against LUAD. Jessica Bockhorn et al. have found that TWF1 has a close association with breast cancer development [28] and miR-30c has been suggested to repress chemotherapy resistance of human breast tumor through modulating TWF1 and IL-11 [25]. Yet, the exact functions of SBF2-AS1, miR-142-3p and TWF1 in pancreatic cancer remains unclear. Therefore, we launched this present study to unearth the role of lncRNA SBF2-AS1 as a sponge of miR-142-3p to modulate TWF1 in the gemcitabine resistance of pancreatic cancer.

## RESULTS

### High expression of lncRNA SBF2-AS1 is found in pancreatic cancer tissues and cells, and mainly located in the cytoplasm

**Figure 1:**
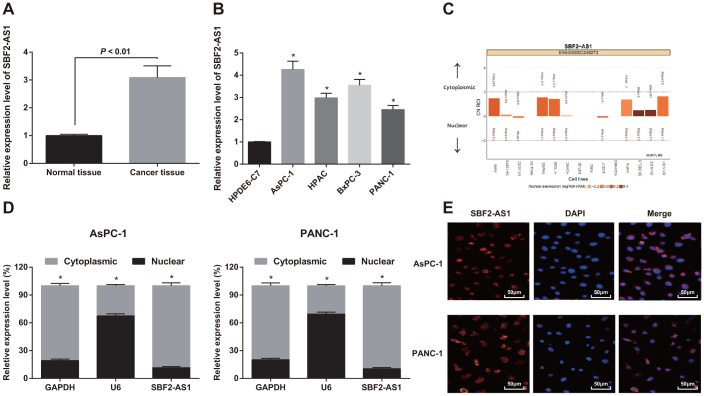
**Expression of SBF2-AS1 in pancreatic cancer tissues and cells.** (**A**) Detection of SBF2-AS1 expression in pancreatic cancer and adjacent normal tissues by RT-qPCR, N = 82. (**B**) Detection of SBF2-AS1 expression in pancreatic cancer cells and normal cells by RT-qPCR. (**C**) Bioinformatics analysis to predict the expression localization of SBF2-AS1. (**D**) Detection of expression localization of SBF2-AS1 by nuclear and cytoplasmic separation assay. (**E**) FISH experiment to verify the expression localization of SBF2-AS1. Repetitions = 3; Data was analyzed using the t test or one-way ANOVA. * *P* < 0.05 vs HPDE6-C7 cells.

SBF2-AS1 expression in pancreatic cancer and adjacent normal tissues was determined by RT-qPCR, and the results showed that the expression of SBF2-AS1 in pancreatic cancer tissues was higher than that in adjacent normal tissues (*P* < 0.01; Figure 1A).

With the average expression of SBF2-AS1 as the critical value, pancreatic cancer patients were assigned into high expression group (≥ 3.09) and low expression group (< 3.09) so as to analyze the relationship between SBF2-AS1 expression and the clinicopathological features and survival prognosis of pancreatic cancer patients. The results revealed that SBF2-AS1 expression was correlated with the degree of differentiation, TNM stage (for observing the total stage of cancer patients) and LNM (an indicator of pathological features) in pancreatic cancer patients. In pancreatic cancer tissues, SBF2-AS1 decreased with the increase of differentiation degree, and SBF2-AS1 expression was higher in patients with III + IV stage than in patients with stage I + II. SBF2-AS1 expression in patients with LNM was higher than that without LNM (all *P* < 0.05). No correlation exhibited between SBF2-AS1 and age, gender and tumor site of pancreatic cancer (all *P* > 0.05; Table 1). In addition, after 6 months follow-up of pancreatic cancer patients, we found that 45 out of 82 pancreatic cancer patients died and 37 survived after 6 months. SBF2-AS1 expression was higher in the death group than in the survival group (*P* < 0.05; Table 2).

**Table 1:** Relationship between the expression of SBF2-AS1 and clinicopathological characteristics in patients with pancreatic cancer [n(%)].

**Clinicopathological characteristic**	**Case**	**SBF2-AS1 expression**		**χ2**	***P***
		**High (n = 39)**	**Low (n = 43)**		
Age (years)				0.58	0.446
< 60	33	14(42.4)	19(57.6)		
≥ 60	49	25(51.0)	24(49.0)		
Gender				2.21	0.138
Male	51	21(41.2)	30(58.8)		
Female	31	18(58.1)	13(41.9)		
Tumor site				1.23	0.544
Caput pancreatitis	52	27(51.9)	25(48.1)		
Corpora pancreatitis	24	10(41.7)	14(58.3)		
Cauda pancreatitis	6	2(33.3)	4(66.7)		
Differentiation degree				4.96	0.026
High	22	6(27.3)	16(72.7)		
Moderate + low	60	33(55.0)	27(45.0)		
TNM stage				7.44	0.006
I + II	36	11(30.6)	25(69.4)		
III + IV	46	28(60.9)	18(39.1)		
Lymph node metastasis				5.39	0.020
With	48	28(58.3)	20(41.7)		
Without	34	11(32.4)	23(67.6)		

**Table 2:** Relationship between SBF2-AS1 expression and survival and prognosis in patients with pancreatic cancer [n(%)].

**State**	**Case**	**SBF2-AS1 expression**		**χ2**	***P***
		**High (n = 39)**	**Low (n = 43)**		
Death	45	27(60.0)	18(40.0)	6.19	0.013
Survival	37	12(32.4)	25(67.6)		

Similarly, RT-qPCR suggested that SBF2-AS1 expression in pancreatic cancer cells (AsPC-1, HPAC, BxPC-3 and PANC-1) elevated relative to normal pancreatic ductal epithelial cells (HPDE6-C7) (all *P* < 0.05). SBF2-AS1 expression in AsPC-1 and PANC-1 cells was maximally and minimally different from that in normal pancreatic ductal epithelial cells (HPDE6-C7), so AsPC-1 and PANC-1 cells were chosen for subsequent experiments (Figure 1B).

SBF2-AS1’s subcellular localization was predicted by bioinformatics website, which suggested that SBF2-AS1 was mainly located in the cytoplasm in tumor cells (Figure 1C).

SBF2-AS1’s subcellular localization in AsPC-1 and PANC-1 cells was also analyzed by nuclear and cytoplasmic separation assay, which found that SBF2-AS1 was expressed in both nucleus and cytoplasm of AsPC-1 and PANC-1 cells, but the expression level in the cytoplasm was higher than that in the nucleus, indicating that SBF2-AS1 is of great significance in pancreatic cancer (Figure 1D).

Furthermore, the expression localization of SBF2-AS1 in AsPC-1 and PANC-1 cells was verified by FISH. The results indicated that SBF2-AS1 was expressed in cytoplasm and nucleus of AsPC-1 and PANC-1 cells, and was mainly positioned in cytoplasm, which was in line with the results of nuclear and cytoplasmic separation assay (Figure 1E).

### High expression of SBF2-AS1 is found in gemcitabine resistant pancreatic cancer cells

**Figure 2:**
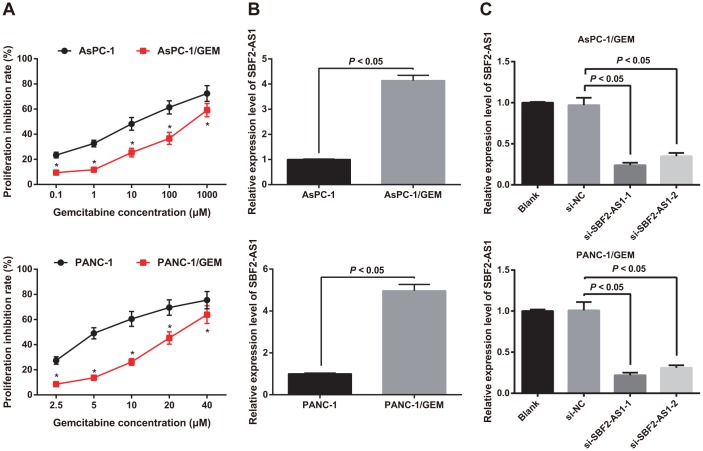
**Identification of drug resistance and expression of SBF2-AS1 in AsPC-1/GEM and PANC-1/GEM cells.** (**A**) Identification of drug resistance in AsPC-1 and PANC-1, AsPC-1/GEM and PANC-1/GEM cells by MTT assay. (**B**) Detection of the expression of SBF2-AS1 in AsPC-1 and PANC-1, AsPC-1/GEM and PANC-1/GEM cells by RT-qPCR. (**C**) RT-qPCR to detect transfection efficiency of SBF2-AS1 siRNA. Repetitions = 3; Data was analyzed using the t test or one-way ANOVA. * *P* < 0.05 vs parent cells with same concentration.

With the aim to verify the drug resistance of drug resistant cell line, the sensitivity of gemcitabine resistant cell lines AsPC-1/GEM and PANC-1/GEM to gemcitabine was detected by MTT assay, and the parental AsPC-1 and PANC-1 cells were used as controls. The findings revealed that the IC50 of AsPC-1 parent cells was 12.45 μM, which was lower than that in AsPC-1/GEM cells (422.68 μM). Similarly, the IC50 of PANC-1 parent cells was lower than that in PANC-1/GEM cells (5.16 μM vs 23.47 μM) (*P* < 0.05; Figure 2A). It indicated that gemcitabine resistant cell lines AsPC-1/GEM and PANC-1/GEM were successfully constructed.

Differential expression of SBF2-AS1 in AsPC-1 and PANC-1 parent cells as well as AsPC-1/GEM and PANC-1/GEM was analyzed by RT-qPCR. It was suggested that SBF2-AS1 expression in AsPC-1/GEM and PANC-1/GEM was higher than that in parental cells AsPC-1 and PANC-1 (all *P* < 0.05) (Figure 2B), which suggested that SBF2-AS1 was related to gemcitabine resistance in pancreatic cancer.

In order to further explore the association between SBF2-AS1 and gemcitabine resistance in pancreatic cancer, we transfected the siRNA-1 and siRNA-2 plasmids of SBF2-AS1 into AsPC-1/GEM and PANC-1/GEM cells to interfere with the expression of SBF2-AS1, which further to detect the changes in SBF2-AS1 expression by RT-qPCR. Relative to the blank group, SBF2-AS1 expression in AsPC-1/GEM and PANC-1/GEM cells transfected with NC sequence did not change (*P* > 0.05), but SBF2-AS1 expression declined in the AsPC-1/GEM and PANC-1/GEM cells transfected siRNA-1 and siRNA-2 plasmids of SBF2-AS1 (both *P* < 0.05; Figure 2C). The results elucidated that the siRNA plasmid of SBF2-AS1 was transfected successfully. Among which, the efficacy of si-SBF2-AS1-1 was better than si-SBF2-AS1-2, so si-SBF2-AS1-1 was selected for subsequent experiments, which was named si-SBF2-AS1.

### Knock-down of SBF2-AS1 inhibits proliferation of gemcitabine resistant pancreatic cancer cells

**Figure 3:**
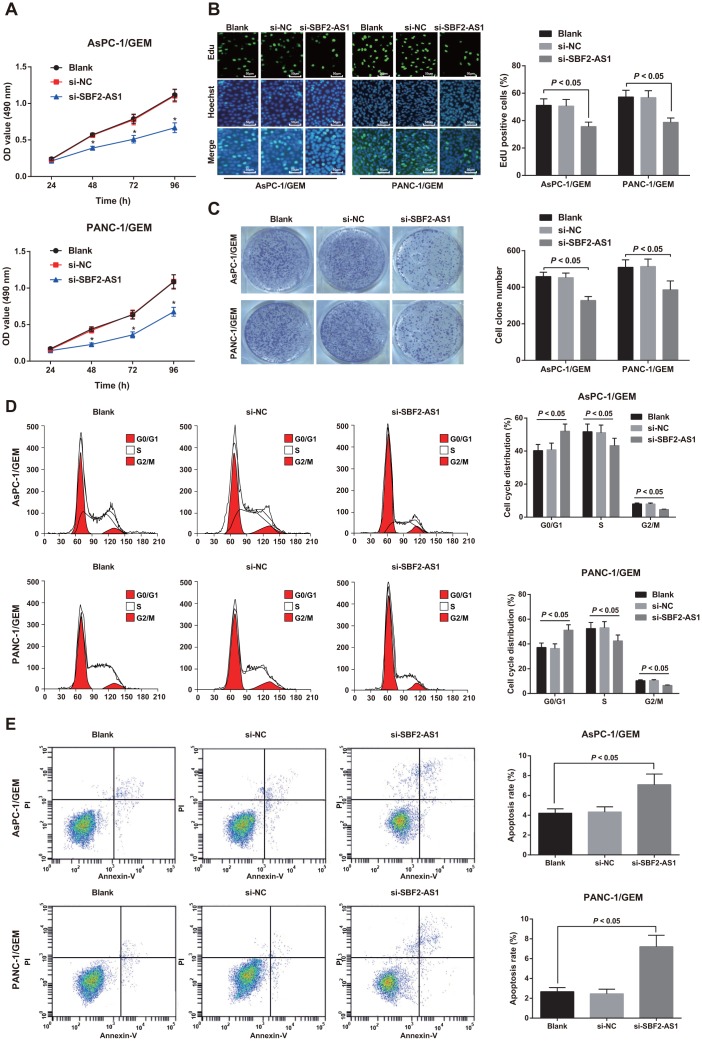
**Effect of knock-down of SBF2-AS1 expression on proliferation, cell cycle distribution and apoptosis of drug-resistant pancreatic cancer cells.** (**A**) Determination of cell proliferation of AsPC-1/GEM and PANC-1/GEM cells by MTT assay. (**B**) EdU assay used to detect DNA replication activity in AsPC-1/GEM and PANC-1/GEM cells (× 200). (**C**) Detection of colony formation ability of AsPC-1/GEM and PANC-1/GEM cells in each group by colony formation experiment. (**D**) Flow cytometry utilized to detect cell cycle distribution of AsPC-1/GEM and PANC-1/GEM cells. (**E**) Flow cytometry was utilized to detect cell apoptosis of AsPC-1/GEM and PANC-1/GEM cells. Repetitions = 3; Data was analyzed using the t test or one-way ANOVA.

MTT, EdU and colony formation assays were performed to elucidate the role of SBF2-AS1 in gemcitabine resistant pancreatic cancer cells, and the results showed that no significant difference was detected in the proliferation rate, DNA replication activity and the colony number of AsPC-1/GEM and PANC-1/GEM cells between the si-NC group and the blank group (all *P* > 0.05). The proliferation rate, DNA replication activity and the colony number of cells in the si-SBF2-AS1 group all declined relative to the blank group (all *P* < 0.05; Figure 3A–3C). These results indicate that knock-down of SBF2-AS1 can decrease the proliferative ability of drug resistant pancreatic cancer cells.

### Knock-down of SBF2-AS1 inhibits cell cycle progression and promotes apoptosis of gemcitabine resistant pancreatic cancer cells

Flow cytometry was utilized to detect cell cycle distribution and apoptosis of AsPC-1/GEM and PANC-1/GEM cells. The results revealed that no change in cell proportion of G0/G1 phase, S phase and G2/M phase as well as apoptosis rate between the blank group and the si-NC group (all *P* > 0.05). In contrast to the blank group, the number of G0/G1 phase cells increased, and the number of cells in S phase and G2/M phase decreased, as well as the apoptosis rate elevated in the si-SBF2-AS1 group (all *P* < 0.05; Figure 3D, 3E).

### Knock-down of SBF2-AS1 inhibits EMT of gemcitabine resistant pancreatic cancer cells

**Figure 4:**
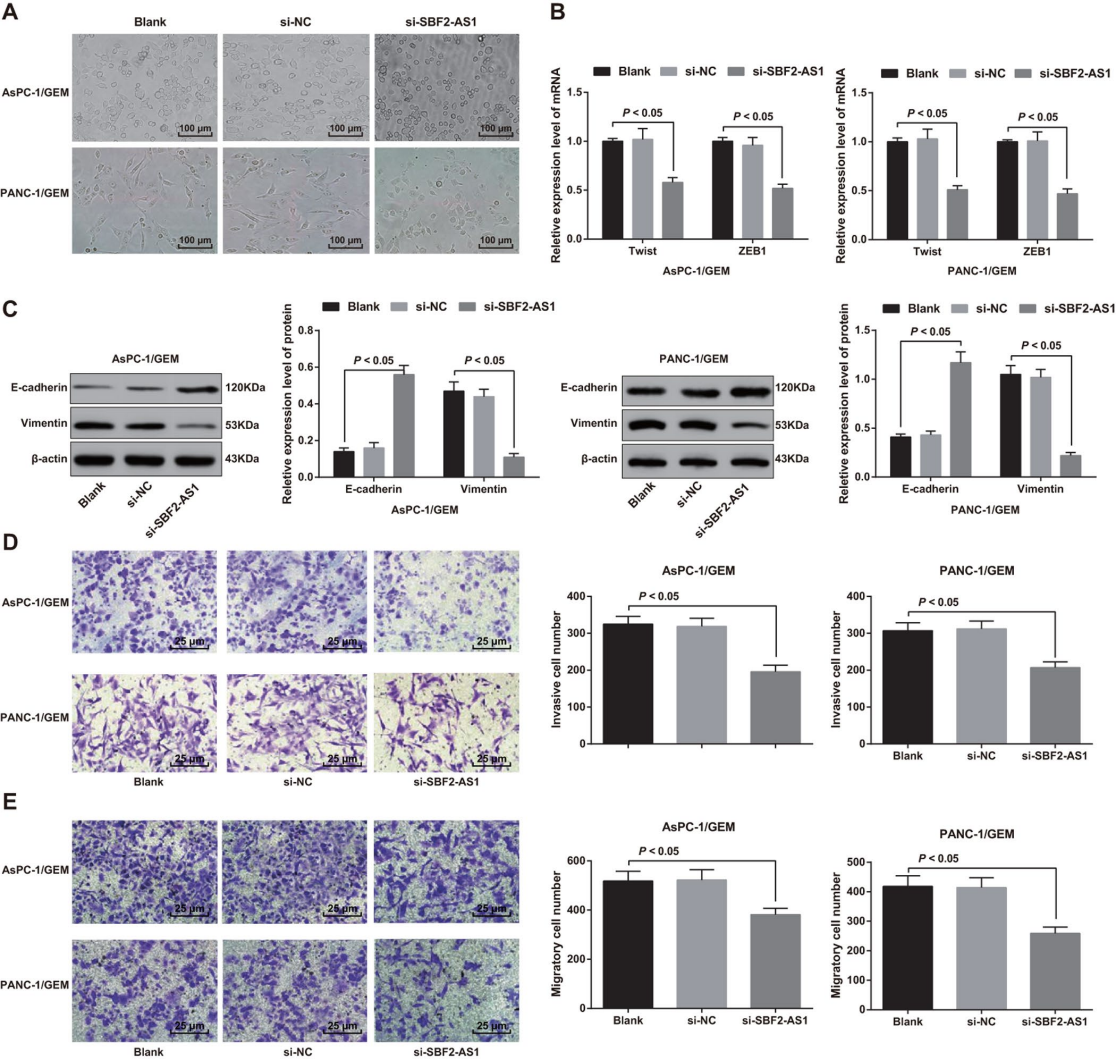
**Effect of knock-down of SBF2-AS1 expression on epithelial-mesenchymal transition, invasion and migration of drug-resistant pancreatic cancer cells.** (**A**) The morphology of cells is observed by an inverted microscope (× 100). (**B**) The mRNA expression of transcriptional factors Twist and ZEB1 in EMT detected by RT-qPCR. (**C**) The expression of E-cadherin and Vimentin detected by western blot analysis. (**D**) The invasion ability of AsPC-1/GEM and PANC-1/GEM cells determined by Transwell assay (× 400). (**E**) The migration ability of AsPC-1/GEM and PANC-1/GEM cells determined by Transwell assay (× 400). Repetitions = 3; Data was analyzed using one-way ANOVA.

In AsPC-1/GEM and PANC-1/GEM cells, transfection of NC sequence in cells had no impact on the morphology of epithelial stroma, while cells introduced with siRNA sequence of SBF2-AS1 suppressed the expression of SBF2-AS1, and drug-resistant pancreatic cancer cells were transformed from fusiform and multi-protuberant mesenchymal morphology to soft circular epithelioid morphology (Figure 4A).

The transcriptional factors (Twist and ZEB1) expression in EMT was detected by RT-qPCR. The results suggested that in AsPC-1/GEM and PANC-1/GEM cells, no difference exhibited in the Twist and ZEB1 mRNA expression between the si-NC group and the blank group (both *P* > 0.05). Twist and ZEB1 mRNA expression in the si-SBF2-AS1 group declined versus that in the blank group (both *P* < 0.05; Figure 4B).

The expression of E-cadherin and Vimentin was determined by western blot analysis. The results indicated that elevated E-cadherin protein and reduced Vimentin protein were found in AsPC-1/GEM and PANC-1/GEM cells in the si-SBF2-AS1 group relative to that in the blank group (both *P* < 0.05; Figure 4C).

### Knock-down of SBF2-AS1 restricts invasion and migration of gemcitabine resistant pancreatic cancer cells

The invasion and migration ability of AsPC-1/GEM and PANC-1/GEM cells was determined by Transwell assay. The number of cell invasion and migration in the blank group was similar to that in the si-NC group (both *P* > 0.05), but the number of cell invasion and migration decreased in the si-SBF2-AS1 group in comparison to that in the blank group (both *P* < 0.05; Figure 4D, 4E). These results suggest that knock-down of SBF2-AS1 suppresses invasion and migration of gemcitabine resistant pancreatic cancer cells.

### Knock-down of SBF2-AS1 increases the chemosensitivity of gemcitabine resistant pancreatic cancer cells

**Figure 5:**
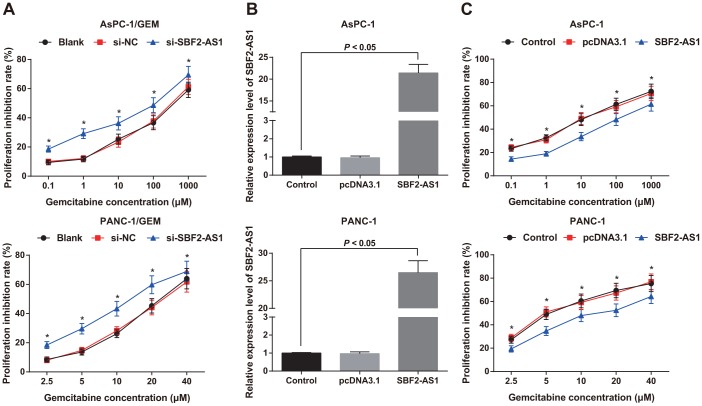
**Chemosensitivity of parental cells and corresponding drug-resistant cells as well as expression of SBF2-AS1 in parental cells.** (**A**) Detection of gemcitabine sensitivity in AsPC-1/GEM and PAN-1/GEM cells by MTT assay. (**B**) Detection of transfection efficiency of SBF2-AS1 overexpression plasmid by RT-qPCR. (**C**) Detection of gemcitabine sensitivity in AsPC-1 and PANC-1 cells by MTT assay. Repetitions = 3; Data was analyzed using the t test or one-way ANOVA. * *P* < 0.05 vs the same concentration of the blank group or the control group.

The chemosensitivity of AsPC-1/GEM and PANC-1/ GEM cells were detected by MTT assay upon treatment of different concentrations of gemcitabine. The findings revealed that the sensitivity of cells to gemcitabine in the si-NC group was similar to that in the blank group (*P* > 0.05), while the sensitivity of the cells to gemcitabine in the si-SBF2-AS1 group was higher than that in the blank group, and the inhibitory rate of gemcitabine elevated at the same concentration on the proliferation of si-SBF2-AS1 cells (all *P* < 0.05; Figure 5A).

The overexpression plasmids of SBF2-AS1 were transfected into AsPC-1 and PANC-1 parental cells. In order to confirm the transfection effect, SBF2-AS1 expression in AsPC-1 and PANC-1 cells was detected by RT-qPCR, which found that SBF2-AS1 expression in AsPC-1 and PANC-1 cells increased significantly after transfection of SBF2-AS1 overexpression plasmids (*P* < 0.05; Figure 5B), which indicated that AsPC-1 and PANC-1 cells expressing SBF2-AS1 were successfully constructed.

The sensitivity of parental AsPC-1 and PANC-1 cells to gemcitabine was also detected by MTT assay, and the findings showed that the inhibitory rate of gemcitabine on the proliferation of cells presented no significant difference in the control group and the pcDNA3.1 group (*P* > 0.05). The inhibition rate of cell proliferation decreased in the SBF2-AS1 group compared to that in the blank group (*P* < 0.05; Figure 5C). It is suggest that knock-down of SBF2-AS1 can increase the chemosensitivity of gemcitabine resistant pancreatic cancer cells, while up-regulating the expression of SBF2-AS1 shows an opposite trend.

### SBF2-AS1 reduces the degree of miR-142-3p dissociation in pancreatic cancer cells by binding to miR-142-3p

**Figure 6:**
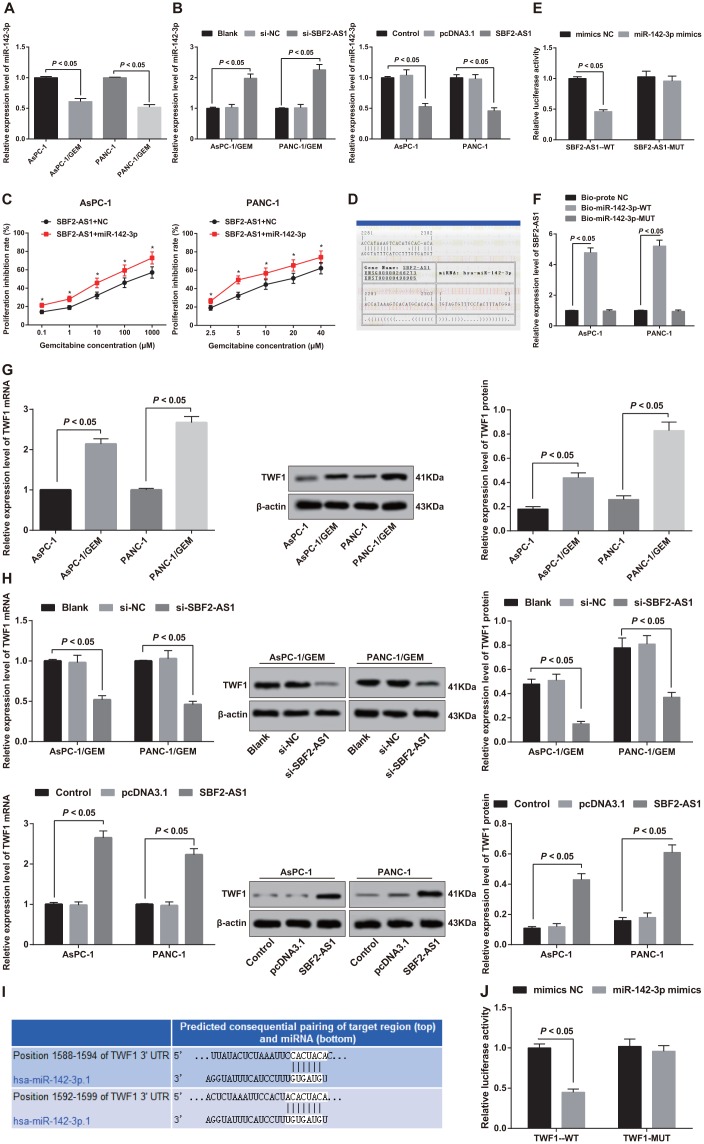
**Analysis of binding relationship between SBF2-AS1 and miR-142-3p, and expression of TWF1 in parent cells and corresponding drug resistant cells and validation of targeting relationship between miR-142-3p and TWF1.** (**A**) Detection of the expression of miR-142-3p in parental and drug-resistant pancreatic cancer cells by RT-qPCR. (**B**) Detection of the expression of miR-142-3p in different cells after intervention of SBF2-AS1 by RT-qPCR. (**C**) Detection of gemcitabine sensitivity in parental cells overexpressing miR-142-3p by MTT assay. (**D**) Bioinformatics website predicts binding sites of SBF2-AS1 and miR-142-3p. (**E**) Dual luciferase reporter gene assay verifies the regulatory relationship between SBF2-AS1 and miR-142-3p. (**F**) RNA-pull down assay to verify the binding relationship between SBF2-AS1 and miR-142-3p in pancreatic cancer cells. (**G**) Detection of TWF1 mRNA and protein expression in parental cells and corresponding drug-resistant cells by RT-qPCR and western blot analysis. (**H**) RT-qPCR and western blot analysis used to detect the expression of TWF1 in transfected cells. (**I**) Bioinformatics software predicts the targeting relationship between miR-142-3p and TWF1. (**J**) Luciferase activity determination validates the targeting relationship between miR-142-3p and TWF1. Repetitions = 3; Data was analyzed using the t test or one-way ANOVA.

MiR-142-3p expression in pancreatic cancer parent AsPC-1 and PANC-1 cells as well as corresponding drug-resistant cells AsPC-1/GEM and PANC-1/GEM was determined by RT-qPCR. The results indicated that compared with parent AsPC-1 and PANC-1 cells, miR-142-3p in the corresponding drug-resistant cells AsPC-1/GEM and PANC-1/GEM decreased significantly (all *P* < 0.05; Figure 6A). It is suggested that SBF2-AS1 is negatively correlated with miR-142-3p in pancreatic cancer resistance.

Further detection revealed that miR-142-3p was increased in AsPC-1/GEM and PAN-1/GEM cells with stable and lowly expressed SBF2-AS1. However, miR-142-3p decreased in parent AsPC-1 and PANC-1 cells stably over-expressing SBF2-AS1 (all *P* < 0.05; Figure 6B). It was confirmed that SBF2-AS1 negatively regulated miR-142-3p in pancreatic cancer resistance.

By co-transfecting pcDNA3.1-SBF2-AS1 and mimics NC (SBF2-AS1 + NC group) and co-transfecting pcDNA3.1-SBF2-AS1 and miR-142-3p mimics (SBF2-AS1 + miR-142-3p group), we compared the sensitivity of parent AsPC-1 and PANC-1 cells to gemcitabine. Versus the SBF2-AS1 + NC group, the sensitivity of cells to gemcitabine at different concentrations increased significantly in the SBF2-AS1 + miR-142-3p group (*P* < 0.05; Figure 6C). It comes to a conclusion that overexpression of miR-142-3p can reverse the upregulation of SBF2-AS1 on promotion of gemcitabine resistance in pancreatic cancer.

Through online analysis software, it was forecasted that there was a specific binding region between SBF2-AS1 gene sequence and miR-142-3p sequence (Figure 6D). The results of dual luciferase reporter gene assay found that in contrast to the mimics NC group, the luciferase activity repressed in the SBF2-AS1-WT + miR-142-3p mimics group (*P* < 0.05), while the luciferase activity did not change significantly in the SBF2-AS1-MUT + miR-142-3p mimics group (*P* > 0.05), indicating that there was a binding site between SBF2-AS1 and miR-142-3p (Figure 6E).

The findings in RNA-pull down assay demonstrated that SBF2-AS1 expression increased in AsPC-1 and PANC-1 cells in the Bio-miR-142-3p-WT group (*P* < 0.05), while no difference exhibited in SBF2-AS1 expression in the Bio-miR-142-3p-MUT group (*P* > 0.05), in comparison to the Bio-probe NC group. Bio-miR-142-3p-WT can promote the enrichment of SBF2-AS1, but Bio-miR-142-3p-MUT cannot. It is confirmed that SBF2-AS1 can reduce the degree of miR-142-3p dissociation in pancreatic cancer cells by binding to miR-142-3p (Figure 6F).

### SBF2-AS1 represses TWF1 by competitively binding with miR-142-3p in pancreatic cancer

Expression of TWF1 in pancreatic cancer parental AsPC-1 and PANC-1 cells and corresponding drug-resistant cells AsPC-1/GEM and PANC-1/GEM were detected. The findings suggested that TWF1 in drug-resistant AsPC-1/GEM and PANC-1/GEM cells was expressed highly than that in parent AsPC-1 and PANC-1 cells (all *P* < 0.05; Figure 6G). Combined with the expression of SBF2-AS1, it is suggested that there may be a positive correlation between SBF2-AS1 and TWF1 in pancreatic cancer.

In order to verify the relationship between SBF2-AS1 and TWF1, we detected TWF1 expression in AsPC-1/GEM and PANC-1/GEM cells with stable and low expression of SBF2-AS1. The results elucidated that TWF1 was decreased simultaneously in AsPC-1/GEM and PANC-1/GEM cells with stable and lowly expressed SBF2-AS1. Similarly, TWF1 was increased in parent AsPC-1 and PANC-1 cells with stable overexpression of SBF2-AS1 (*P* < 0.05; Figure 6H), which indicated that SBF2-AS1 could positively modulate TWF1 in pancreatic cancer.

The targetscan.org predicted that there was a targeting relationship between miR-142-3p and TWF1 (Figure 6I). The results of luciferase activity found that the relative luciferase activity inhibited in 293T cells co-transfected with TWF1-WT and miR-142-3p mimics (*P* < 0.05), but the cells co-transfected with TWF1-MUT and miR-142-3p mimics did not affect the relative luciferase activity *(P* > 0.05; Figure 6J), suggesting TWF1 is a direct target gene of miR-142-3p. Therefore, combined with the above confirmed binding relationship between SBF2-AS1 and miR-142-3p, we believe that SBF2-AS1 can inhibit the expression of TWF1 by competitively binding with miR-142-3p in pancreatic cancer, thereby affecting gemcitabine resistance in pancreatic cancer.

## DISCUSSION

Evidences have found out many susceptibility loci for pancreatic cancer [29–31], while the mechanism is not well elucidated. Inspiringly, it has been demonstrated that lncRNAs are significant factors in pancreatic cancer, and some lncRNAs may even become potent biomarkers or drug targets for pancreatic cancer [32]. Based on the progress of molecular biological research on pancreatic cancer, new therapeutic strategies capable of interrupting aggressive tumor progression seem to hold the greatest promise [33]. Therefore, we carried out this current study to figure out the role of lncRNA SBF2-AS1 as a ceRNA to sponge miR-142-3p in regulating TWF1 in the gemcitabine resistance of pancreatic cancer. Collectively, this present study suggests that suppressed SBF2-AS1 restricts TWF1 expression by sponging miR-142-3p to promote gemcitabine resistance in pancreatic cancer.

First of all, this study found that SBF2-AS1 was upregulated in pancreatic cancer tissues and cells. A study has demonstrated that SBF2-AS1 is upregulated in ESCC, and SBF2-AS1 reduction resulted in the suppression of the ESCC cell proliferative and invasive ability [12]. Another study has revealed that silencing of SBF2-AS1 also blocked proliferation ability of NSCLC cells [10]. In addition, this study also found that the knock-down of SBF2-AS1 led to repressed proliferation, EMT, invasion and migration as well as promoted apoptosis of gemcitabine resistant pancreatic cancer cells. Meanwhile, lncRNAs are able to regulate tumor proliferation, migration as well as metastasis through controlling transcription, epigenetic modification, alternative splicing and protein translation [34]. Some recent articles have concentrated on the functions of lncRNAs in carcinogenesis and cancer progression in cancers, including pancreatic cancer. Liu and his colleagues reported an upregulation of lncRNA MALAT1 in PDAC in contrast to adjacent normal tissues [35]. Another article also suggested that HOTAIR presented an elevated expression in pancreatic tumors [36]. Besides, it has been found that the overexpression of PVT1 contributed to inhibited gemcitabine sensitivity in the pancreatic cancer [37]. Furthermore, Jiao and his colleagues found that MALAT-1 is able to decrease gemcitabine chemosensitivity in AsPC-1 and CFPAC-1 cell lines [38].

In addition, this present study indicated that SBF2-AS1 inhibited the expression of TWF1 by competitively binding with miR-142-3p in pancreatic cancer. Generally, lncRNAs experience changes in different cancer cells, thus having a great impact on lncRNA-miRNA and protein interactions [39, 40]. Wu et al. have stated that miR-142-3p restricts the migration and invasion of hepatocellular carcinoma cells by directly and negatively regulates RAC1, which highlights the importance of miRNAs in tumorigenesis [41]. Another study has suggested that miR-142-3p resulted in the inhibited proliferation and invasion of cervical cells through directly regulating FZD7 expression [42]. It has been also found out that miR-30c inhibits human breast tumor chemotherapy resistance through regulating TWF1 [25]. The aforementioned studies have verified miR-142-3p might function in cancer progression through targeting some genes, and TWF1 might also exert functions in cancer progression after mediated by some miRNAs. Li et al. have reported that hypoxia-induced lncRNA NUTF2P3-001 induced cell proliferation in pancreatic cancer cells by cabsorbing miR-3923 to regulate KRAS [43]. Importantly, a previous study has suggested that HOTTIP promoted gemcitabine resistance through the regulation of HOXA13, indicating that HOTTIP and HOXA13 may be potential therapeutic target and molecular biomarker for pancreatic cancer [44]. Gao et al. found that the ROR sponged miR-145, thereby activating the derepression of Nanog with the aim to induce cell proliferation and carcinogenesis in pancreatic cancer cells [45].

In summary, we have identified that SBF2-AS1 is up-regulated in pancreatic cancer. Meanwhile, the value of SBF2-AS1 as a potential prognostic and/or therapeutic biomarker in pancreatic cancer was supported by findings in this present study that the epigenetic mechanism of the competitive inhibition of SBF2-AS1 or TWF1 expression by miR-142-3p for inhibiting proliferation, EMT, invasion and migration and promoting apoptosis of gemcitabine resistant pancreatic cancer cells (Supplementary Figure 1). Furthermore, knock-down of SBF2-AS1 can enhance the negative regulation of TWF1 by miR-142-3p and effectively reduce the malignant tumor characteristics of pancreatic cancer.

## MATERIALS AND METHODS

### Ethics statement

The study was permitted by the independent ethics committee in Fudan University Shanghai Cancer Center with the ethical principles in the Declaration of Helsinki. All the patients offered written informed consent before the study.

### Study subjects

From June 2015 to January 2017, 82 pancreatic cancer patients diagnosed and treated in Fudan University Shanghai Cancer Center were selected for surgical resection of pancreatic cancer tissues and their adjacent normal tissues (at least 2 cm above the margin of the tumor). Patients were enrolled into our study if they fit with these criteria: all patients underwent radical surgical resection and were diagnosed as pancreatic cancer by pathology; patients did not receive preoperative chemotherapy, radiotherapy, or other clinical adjuvant therapy before operation; the survival of patients was expected to be longer than 3 months. The patients were removed from our study if they were combined with other malignant tumors; they were pregnant or lactating women; patients were combined with severe dysfunctions of heart, liver, lung, kidney, or blood system. The clinicopathological data of the patients were collected in detail, including age, gender, tumor location, degree of differentiation, tumor node metastasis (TNM) stage as well as lymph node metastasis (LNM). Meanwhile, pancreatic cancer patients were followed up for 6 months to record survival and death.

### Cell selection and culture

Pancreatic cancer cell lines including AsPC-1, HPAC, BxPC-3, and PANC-1 together with normal pancreatic ductal epithelial cell line HPDE6-C7 were acquired from ATCC (Rockefeller, Maryland, USA). All kinds of cells were cultured in a 37°C with 5% CO_2_ incubator in RPMI1640 medium supplemented with 10% fetal bovine serum (FBS, both from Gibco, Grand Island, NY, USA). After 48 hours, the cells were detached and then subcultured. The logarithmic growth phase cells were taken for subsequent experiments.

The pancreatic cancer cells were selected and AsPC-1/GEM and PANC-1/GEM (gemcitabine resistant cell lines) were induced by intermittent concentration increase [46]. The AsPC-1 and PANC-1 cells were cultivated with gemcitabine in different concentrations for 1 w. The cell death conditions were checked and the median lethal dose (LD80) concentration was chosen as the initial concentration for cultivating the resistant cells. Next, cells were cultured for 48 h in this medium, and then fostered in drug-free RPMI-1640 medium. The cells were passaged two times when the cells entering into the logarithmic growth phase, which were then exposed to gemcitabine in double LD80 concentration. With nine concentration gradients and more than ten months of cultivation, the cells were cultured in drug-free RPMI-1640 medium for 2 months.

### Cell treatment

Thedrug-resistant cell lines AsPC-1/GEM and PANC-1/GEM were grouped into blank group (cells without any treatment), si-negative control (NC) group (cells introduced with NC plasmid), and si-SBF2-AS1 group (cells introduced with SBF2-AS1 siRNA plasmid).

The parent AsPC-1 and PANC-1 cells were obtained and then grouped into control group (cells with no treatment), pcDNA3.1 group (cells transducted with pcDNA3.1 plasmid), SBF2-AS1 group (cells transducted with pcDNA3.1-SBF2-AS1 plasmid), SBF2-AS1 + NC group (cells transducted with pcDNA3.1-SBF2-AS1 and mimics NC plasmid), and SBF2-AS1 + miR-142-3p group (cells transducted with pcDNA3.1-SBF2-AS1 and miR-142-3p mimics plasmid). SBF2-AS1 siRNA-1 and siRNA-2 (the siRNA sequences for SBF2-AS1 were si-SBF2-AS1-1: (Sense) 5′-CAGAAGGAGUCUACUGC UAAG-3′ and (Antisense) 5′-UAGCAGUAGACUCC UUCUGGG-3′ and si-SBF2-AS1-2: (Sense) 5′-GCAA GCCUGCAUGGUACAUTT-3′ and ’ (Antisense) 5′-AU GUACCAUGCAGGCUUGCTT-3 and NC plasmid, pcDNA3.1, pcDNA3.1-SBF2-AS1, mimics NC and miR-142-3p mimics were constructed by Sangon BiotechCo., Ltd., (Shanghai, China). Cell transfection was conducted in the light of the requirements of Lipofectamine 2000 (Invitrogen, Carlsbad, CA, USA), and then detected 48 hours later.

### Reverse transcription quantitative polymerase chain reaction (RT-qPCR)

The Trizol method (Invitrogen, Carlsbad, CA, USA) was implemented for the extraction of the total RNA in both cells and tissues. The complementary DNA (cDNA) was acquired by avian myeloblastosis virus reverse transcriptase after obtaining l μg RNA. SYBR Green was used for qPCR, and U6 (nuclear RNA and miR-142-3p) and glyceraldehyde phosphate dehydrogenase (GAPDH) was selected as loading controls. The primer sequences of genes were designed and synthesized by Shanghai Genechem Co., Ltd. (Shanghai, China) (Table 3). Real-time fluorescence quantitative PCR instrument (ABI 7500, ABI, Foster City, CA, USA) was adopted for detection. The 2^-ΔΔCt^ method was used to analyze the expression of genes.

**Table 3:** Primer sequence.

**Gene**	**Primer sequence**
SBF2-AS1	F: 5′- AGTTGAGGGTCAAGCTGCTC-3′
	R: 5′- TAGAGAGCCAGGGGATG-3′
miR-142-3p	F: 5′- TGTAGTGTTTCCTACTTTAT-3′
	R: 5′- GTCGTATCCAGTGCAGGG-3′
U6	F: 5′-CGCTTCGGCAGCACATATAC-3′
	R: 5′- TTCACGAATTTGCGTGTCAT-3′
Twist	F: 5′- TCAGCCACTGAAAGGAAAGG-3′
	R: 5′- GTTTTGCAGGCCAGTTTGAT-3′
ZEB1	F: 5′- AAGTGGCGGTAGATGGTAATGT-3′
	R: 5′- AAGGAAGACTGATGGCTGAAAT-3′
TWF1	F: 5′- CACTGACTGCAGCTGAGGAA-3′
	R: 5′- TACATCCCAAGCAGCATGCA-3′
GAPDH	F: 5′- TGGGTGTGAACCATGAGAAG-3′
	R: 5′- GTGTCGCTGTTGAAGTCAGA-3′

Note: F, forward; R, reverse; GAPDH, glyceraldehyde phosphate dehydrogenase.

### Western blot analysis

After obtaining the proteins from tissues and cells, their concentrations were determined in the light of the bicinchoninic acid assay (Boster Biological Technology, Ltd., Wuhan, China). After 10% sodium dodecyl sulfate polyacrylamide gel electrophoresis (Boster Biological Technology, Ltd., Wuhan, China) for protein separation, they were transferred onto polyvinylidene fluoride membrane, followed by blocking with 5% bovine serum albumin for 1 h. After that, the membranes were probed with the primary antibodies to E-cadherin, Vimentin, TWF1 (1 : 1000) and β-actin (internal control, ab154725, 1 : 3000) (both from Abcam, Cambridge, MA, USA). The corresponding secondary antibodies (Miaotong Biotechnology Co., Ltd., Shanghai, China) were appended for 1-h incubation. A enhanced chemiluminescence solution and Gel Doc EZ imager (Bio-Rad, Hercules, CA, USA) were used for developing. The gray value analysis of target band was processed by Image J software (National Institutes of Health, Bethesda, Maryland, USA).

### MTT assay

AsPC-1/GEM and PANC-1/GEM cell suspensions were diluted with an amount concentration and then seeded into 96-well plates with 5 × 10^4^ cells/well. Six parallel wells were set in each group. When reaching 80% confluence, the cells were grouped based upon the above experiments. The cells were next cultured in 20 μL MTT solution (5 mg/mL, Sigma, St. Louis, MO, USA) for 24 h, 48 h, 72 h and 96 h, and incubated at 37°C for 4 h. MTT solution was discarded and dimethyl sulfoxide (DMSO, Sigma, St. Louis, MO, USA) was supplemented into each well. The optical density (OD) value in each well was measured at 490 nm by a microplate reader.

After detaching the untransfected and transfected parent AsPC-1 and PANC-1 cells as well as the corresponding drug-resistant cells AsPC-1/GEM and PANC-1/GEM with 0.25% trypsin, the cell detachment was stopped in the medium and the cells were counted after centrifugation. Different cells were inoculated to 96-well plates according to the number of 3000 - 5000 cells per well, and the cell suspension of each well was 100 μL. After the cells reached upon 70-80% confluence, the cells were replaced with serum-free RPMI 1640 culture solution for 24 h, and the cells were synchronized. AsPC-1 and AsPC-1/GEM cells were treated with a concentration gradient of 0.1, 1, 10, 100, and 1000 μM, respectively. PANC-1 and PANC-1/GEM cells were treated with a concentration gradient of 2.5, 5, 10, 20 and 40 μM, respectively. After 72 hours of treatment, the cells were treated with 20 μL MTT solution at 37°C for 4 h, and the OD value of each well was measured at 490 nm. Cell proliferation inhibition rate and 50% inhibitory concentration (IC50) were calculated

### EdU assay

The cell-light EdU luminescence assay kit (RiboBio, Guangzhou, China) was employed to detect the DNA replication ability of cells. After routine treatment of AsPC-1/GEM and PANC-1/GEM cells in each group, the cells were inoculated in 96-well plates at 1.0 × 10^4^ cells/well, with three parallel wells set in each group. Afterwards, the cells were fostered with 100 μL 50 μM EdU solution for 2 h, fastened with 4% paraformaldehyde, treated with 2% glycine, and permeabilized with 150 μL permeating agent. According to the requirements of EdU kit, the cells were continually treated. Under a fluorescence microscope (FSX100, Olympus, Tokyo, Japan), 5 visual fields were selected in a random fashion. The blue fluorescence corresponded to all the cells, and the red fluorescence reflected the EdU-infiltrated replicating cells. The rate of EdU positive cells was counted.

### Colony formation assay

The AsPC-1/GEM and PANC-1/GEM cells in each group were dispersed and seeded with 200 cells into six-well plates. The cells were then dispersed again and cultured for two or three weeks. When the cell colony was seen, the culture was ended, and the culture solution was removed and fastened with 4% paraformaldehyde. After that, the cells were stained with Giemsa application solution, and then removed slowly by water. The number of cell colony was viewed under a microscope.

### Flow cytometry

#### Cell cycle distribution

Thee AsPC-1/GEM and PANC-1/GEM cells were cultured with 5% CO_2_ at 37°C. When reaching 80% confluence, cells were replaced with serum-free RPMI1640 medium and synchronize starved for 24 h [47]. Then, after treating the cells in the above-mentioned experimental groups, the cells were cultured until the time required for the experiment. The AsPC-1/GEM and PANC-1/GEM cells were collected and centrifuged to remove the supernatant. Afterwards, the cells were appended to make the cell concentration to 1 × 10^6^ cells/mL, thus the single cell suspension was prepared, which was centrifuged at 2000 rpm for 5 min to remove the supernatant. The 70% ethanol (500 μL) was appended to each group, and then fixed for 2 h. The fixative solution was removed, and 1 mL PBS was appended to eluate fixation solution, and centrifuged at 2000 rpm for 3min to remove the supernatant. Afterwards, the cells were appended with 100 μL RNase A for 30 min, 400 μL PI and mixed for 30 min devoid of light. The red fluorescence at the 488 nm excitation wavelength was recorded.

#### Cell apoptosis

The AsPC-1/GEM and PANC-1/GEM cells were harvested and centrifuged for discarding the supernatant. Next, the suspension cells were centrifuged to collect the cells, and the cell concentration was altered to 1 × 10^6^ cells/mL, and 200 μL cells were rinsed with 1 mL pre-cooled PBS two times and then centrifuged. The cells were suspended in binding buffer (100 μL) and 2 μL Annexin-V-FITC (20 μg/mL), gently mixed, and placed on the ice. Next, the cells were put to a flow detection tube, supplemented with 300 μL PBS, and added with 1 μL PI (50 μg/mL) to each sample before putting on the machine, and the detection was lasted for 30 min. The AnnexinV-PI double negative group (unstained cells), the AnnexinV-single staining group (AnnexinV-FITC-stained cells alone) and the PI-single staining group (PI stained cells alone) were set to be used as a reference for fluorescence compensation adjustment. Flow cytometer detection: the excitation wavelength set as 488 nm and the emission wavelength set as 530 nm. FL1 is the FITC channel of AnnexinV-FITC green fluorescence; FL2 is the PI channel of PI red fluorescence.

Annexin V was considered as horizontal axis, while PI as longitudinal axis; left upper quadrant (Annexin V-, PI+) as necrotic cells, left lower quadrant (Annexin V-, PI-) as living cells, right upper quadrant (Annexin V+, PI-) as early apoptotic cells and right lower quadrant (Annexin V+, PI+) as late apoptotic cells.

### Transwell assay

The AsPC-1/GEM and PANC-1/GEM cells were detached in cells of each group, and 1 × 10^5^ cells were incubated with Transwell chamber coating with matrigel (80 μL, Becton, Dickinson and Company, Franklin lake, New Jersey, USA), and 100 μL serum-free DMEM was also appended. The basolateral chamber was appended with complete medium, and the cells of the apical chamber were removed by cotton swabs with 24-h incubation. Then cells were fastened in 4% paraformaldehyde and dyed by crystal violet solution. Five fields of view were selected in a random fashion for photographing under a microscope. The number of cells penetrating the membrane was counted.

### Fluorescence in situ hybridization (FISH) assay

First, the bioinformatics website http://lncatlas.crg.eu/ was used to forecast the subcellular localization of SBF2-AS1. Then the nuclear and cytoplasmic separation experiments were carried out with PARISTM Kit (Ambion, Austin, Texas, USA). The cells (10^7^) were added with 500 μL cell fractionation buffer for cell suspension. The supernatant (cytoplasm) and precipitation (nucleus) were separated. The segregated supernatant was preheated with equal volume of 2 × Lysis/Binding Solution and then fully blended to prevent RNA degradation. RNA on the filter membrane was harvested and centrifuged for 30 s to dissolve cytoplasmic RNA. The nucleus precipitation was processed according to the above steps, and the dissolved collections were nucleus RNA. Cytoplasmic and nuclear RNA were collected and retrieved by M-MLV kit. The expression of SBF2-AS1 in nucleus and cytoplasm was detected by RT-qPCR.

Subcellular localization of SBF2-AS1 was verified by FISH assay. Cells were seeded into 24-well plates containing glass slides by 3 × 10^4^ per well. Then, cells were fastened with 100 μL 4% paraformaldehyde for 10 min and then appended with 100 μL 0.5% Triton X-100. Next, prehybridization solution and hybridization solution were supplemented and kept at 37°C for 20 min. The pre-hybridization solution was lightly sucked with the gunhead, and the lncRNA-SBF2-AS1 FISH probe (Ribo, Guangzhou, China) and the hybridization solution was diluted at a ratio of 1 : 50. At the next day, 4 × SSC, 2 × SSC and 1 × SSC were preheated at 50°C, and the cells were rinsed for three times in the order of high concentration to low concentration. After removing SCC, each well was appended with 10 μL DAPI working solution and stand for 8 min, and the sealed. Finally, five different visual fields were observed and captured under a fluorescence microscope (Olympus, Tokyo, Japan).

### Dual luciferase reporter gene assay

Bioinformatics software https://cm.jefferson.edu/rna22/ Precomputed/ was used to forecast the binding site between SBF2-AS1 and miR-142-3p. The binding site between SBF2-AS1 and miR-142-3p was confirmed by luciferase activity assay. The synthetic SBF2-AS1 3′UTR gene fragment was also inserted into pMIR-reporter with the application of endonuclease sites Bamh1 and Ecor1 (Huayuyang Biotechnology, Beijing, China). Meanwhile, the wild type (WT) of SBF2-AS1 was also was designed. The target fragment was infixed into the pMIR-reporter plasmid by way of restriction endonuclease digestion and T4 DNA ligase. The WT and mutant type (MUT) with correct sequence were cotransfected with miR-142-3p mimics and its control into 293T cells (Beinuo Biotechnology, Shanghai, China). After 48-h transfection, the cells were harvested and then lysed. Luciferase activity was evaluated with luciferase detection kit (BioVision, San Francisco, CA, USA) by the way of luminometer (Glomax20/20, Promega, Madison, Wisconsin, USA).

Bioinformatics software http://www.targetscan.org was implemented to forecast the binding site of miR-142-3p to TWF1 3′UTR. TWF1 3′UTR wild type plasmid (TWF1-WT) and mutant type plasmid TWF1 3′UTR (TWF1-MUT) were constructed. The procedure was performed in the light of the plasmid extraction kit (Promega, Madison, Wisconsin, USA). The cells were seeded into 96-well plates and then transfected with Lipofectamine 2000 when reaching about 70% confluence. TWF1-WT and TWF1-MUT were co-transfected into 293T cells after mixed with mimics NC and miR-142-3p mimics respectively. Cells were harvested and lysed after 48 h transfection. The luciferase activity was determined with a luciferase detection kit.

### RNA-pull down assay

Cells were transducted with Biotin-labeled miR-142-3p WT and biotinylated miR-142-3p MUT plasmids (50 nM each). After 48-h transfection, the cells were harvested and incubated with a specific cell lysate (Ambion, Austin, Texas Stilla, USA). Meanwhile, 50 mL cell lysate was appended. The residual pyrolysis was cultured with M-280 streptavidin magnetic beads precoated with RNase-free and yeast tRNA (Sigma, St. Louis, MO, USA). Then it was rinsed two times with cold cracking solution, low salt buffer (three times) and high salt buffer (one time). Antagonistic miR-142-3p probe was set as a NC. Total RNA was extracted by Trizol and SBF2-AS1 expression was determined by RT-qPCR.

### Statistical analysis

All statistical analyses were processed using the SPSS 21.0 software (IBM SPSS, Inc., Chicago, IL, USA). The data were normally distributed by Kolmogorov-Smirnov test. The measurement data were depicted as mean ± standard deviation. The t test was utilized for the two-group comparisons, and one-way analysis of variance (ANOVA) was utilized for the multi-group comparisons. After ANOVA analysis, the Fisher’s least significant difference t test (LSD-t) was conducted for pairwise comparison. *P* values ≤ 0.05 were considered statistically significant.

## Supplementary Material

Supplementary Figure
