# Supplementary material for: Long non-coding SBF2-AS1 acting as a competing endogenous RNA to sponge microRNA-142-3p to participate in gemcitabine resistance in pancreatic cancer via upregulating TWF1
Source: Aging (Albany NY). 2019 Oct 31;11(20):5579–92. doi: 10.18632/aging.102307 (PMC6834408; doi:10.18632/aging.102307)
Supplement: Supplementary Figure [file aging-11-102307-s001.pdf]

SUPPLEMENTARY FIGURE

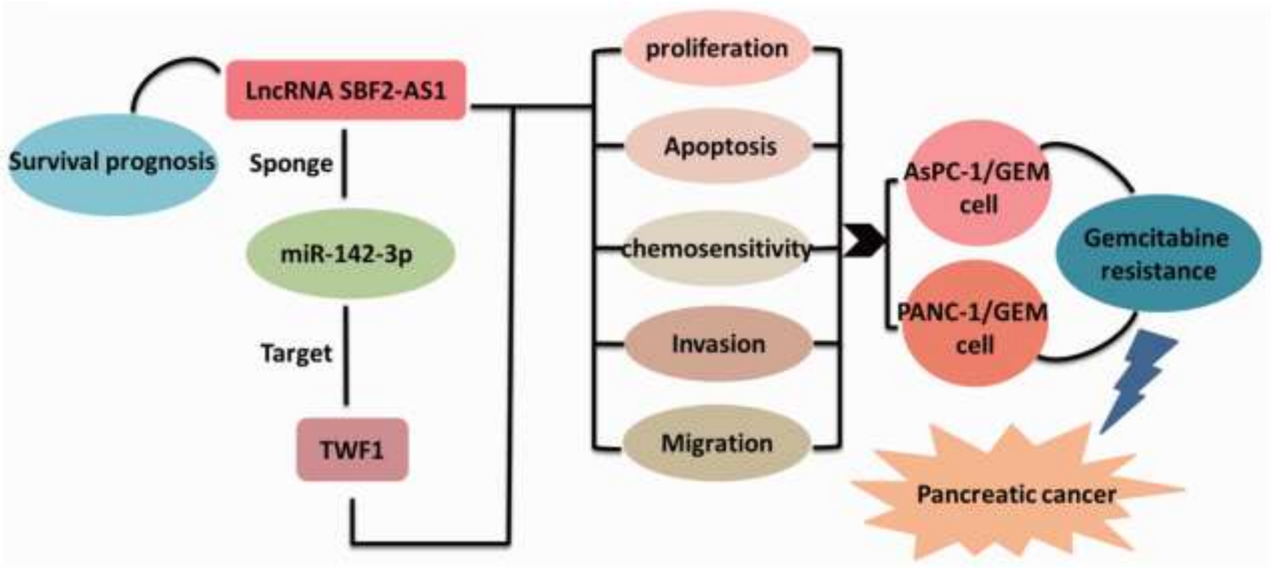

Supplementary Figure 1. The schematic diagram of lncRNA SBF2-AS1/miR-142-3p/TWF1 in pancreatic cancer. LncRNA SBF2-AS1, as a ceRNA of miR-142-3p to regulate TWF1, is involved in the mechanism of gemcitabine resistance in pancreatic cancer.
